# Relationship between the Tensile Properties and Damping Capacity of Fe-22%Mn-12%Cr-4%Co-3%Ni-2%Si Alloys by Fatigue Stress

**DOI:** 10.3390/ma14237160

**Published:** 2021-11-24

**Authors:** Jae-Hwan Kim, Myong-Soo Lee, Jong-Sig Kim

**Affiliations:** 1 Rokkasho Fusion Institutes, Fusion Energy Directorate, National Institutes for Quantum Science and Technology, Rokkasho, Aomori 032-3212, Japan; 2 In-Shop Construction Assistance Department, Hyundai Heavy Industries, Ulsan 44027, Korea; lmslove80@naver.com; 3 Department of Robot-Special Welding, Korea Polytechnics, Chungju 27324, Korea; kjsig@kopo.ac.kr

**Keywords:** fatigue stress, damping capacity, tensile properties, fatigue damage, damping alloy

## Abstract

The relationship between the tensile properties and damping capacity of fatigue-damaged Fe-22%Mn-12%Cr-4%Co-3%Ni-2%Si alloy under various magnitudes of fatigue stress was investigated. Analytical results show that α′- and ε-martensite were formed due to fatigue stress. The formed α′- and ε-martensite followed a specific orientation and surface relief and intersected with each other. TEM observation and pattern analysis reveal that both α′- and ε-martensites formed on the austenite. As a result of X-ray diffraction, with an increase in fatigue stress, the volume fractions of α′- and ε-martensite were increased, and the increasing rate of the volume fraction of α′-martensite was higher than that of the ε-martensite. As the fatigue stress increased, the tensile strength and damping capacity increased, but the elongation decreased. Besides, as the strength increased and the elongation decreased, the damping capacity decreased. This result is inconsistent with the general tendency for metals but similar to that of alloys undergoing deformation-induced martensite transformation.

## 1. Introduction

Since noise and vibration cause many human and material losses, social regulation on these problems has strengthened, and there is an increasing need to reduce and repress noise and vibration for the stability and long lifecycle of machines and equipment.

Damping technology includes systematical, structural, and material damping. Among them, damping technology using high internal friction in materials has the highest effect on damping capacity and excellent machinability. Thus, Kang et al. [1,2,3,4,5] reported that damping alloys concerning strength and damping capacity in Fe-Mn alloys [1,2,3] and stainless steels [4,5], revealing that those depend on the phase fraction.

Damping alloys are materials that can quickly transform external vibration to thermal or other forms of energy [6]. Generally, good damping materials exhibit low machinability, frequency by damping, and are temperature dependent, whereas high strength results in low damping capacity [2]. Although alloys with high damping capacities have been developed and used as vibration sources, the mechanical strength and damping capacity of such alloys vary with the deformation-induced martensite transformation behavior due to different loads and time applied during cyclic loadings [7]. Specifically, it is well known that martensite transformation behavior is associated with strength and damping capacity. It is noted that there is controversy over the nature of martensitic transformation behavior in Fe-Mn alloy systems [8,9]. Furthermore, few studies on the relationship between strength and damping capacity in the damping alloys were found. Thus, it is necessary to obtain various databases under various conditions to develop damping alloys with an excellent combination of strength and damping capacity and securing stability in use.

In this study, to investigate the relationship between the strength and damping capacity of damping alloys by solid solution strengthening and formation of stacking fault, we designed Fe-22%Mn-12%Cr-4%Co-3%Ni-2%Si and investigated the strength and damping capacity of the alloys after fatigue under different stress magnitudes.

## 2. Materials and Methods

Specimens were fabricated as an ingot by melting using a high-frequency vacuum furnace (Auto Tech, Busan, Korea) with the chemical composition shown in Table 1. Solid solution treatment at 1050 °C for 1 h for the specimens was conducted after hot rolling at 1250 °C and downsizing to 20 mm in thickness. The tensile strength, yield strength, and elongation of the specimen in this study are 705 MPa, 353 MPa, and 35%, respectively.

Based on ASTM E8, fatigue damage tests were performed using a tensile specimen with a gauge length of 50 mm. The specimens were fractured at a frequency of 10 Hz and 5 × 10^5^ cycles when the 30%, 50%, and 70% of the yield strengths (i.e., 105, 175, and 245 MPa, respectively) were applied. The stress ratio *R* = 0.1.

The microstructure of each specimen was observed by optical microscope (OM, Olympus, Shinjuku, Japan) after etching with an etching solution (5% hydrochloric acid (HCl), 5% nitric acid (HNO_3_), and 90% methyl alcohol (CH_3_OH)), scanning electron microscope (SEM, S-2400, HITACHI, Tokyo, Japan), and transmission electron microscope (TEM, JEM-2010. 200 kV, JEOL, Tokyo, Japan). After jet polishing of the specimen (3 mm in diameter and 80 μm in thickness), it was observed by TEM with 200 kV of voltage. The volume fractions of each phase with γ (111), ε (002), and α’ (110) in the specimens were measured by X-ray diffraction (D/Max-IIA, Rigaku, Tokyo, Japan) with Mo-Kα in a range of 10°–80° with a speed of 1 degree/min and calculated based on [10,11,12].

Tensile tests were conducted using a 2 mm/min crosshead speed with specimens fatigue-damaged under different fatigue stress amplitudes. To measure the damping capacity, 120 × 10 × 2 mm specimens were fabricated by discharging machining. An internal friction measurement machine (IFT-1500, Ulvac, Kanagawa, Japan) was employed to evaluate the logarithmic decay rate. The wave number was measured to calculate the logarithmic decay rate expressed as *δ* = 1/*n* ln*A0*/*An*, where *n* is the wave number, *A0* the amplitude of the first wave, and *An* the amplitude of the nth wave.

## 3. Results and Discussion

**Figure 1:**
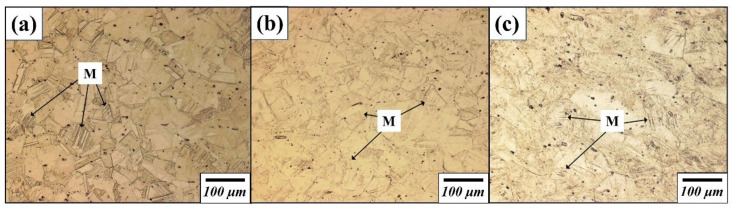
Optical microstructure of damaged Fe-22%Mn-12%Cr-4%Co-3%Ni-2%Si damping alloy under (**a**) 0, (**b**) 105, and (**c**) 245 MPa fatigue stress.

Figure 1 shows the optical microstructure of Fe-22%Mn-12%Cr-4%Co-3%Ni-2%Si damping alloys damaged under different fatigue stresses. The specimens were subjected to fatigue cycles at a frequency of 10 Hz and stress ratio *R* of 0.1 to investigate the variation of the microstructure with the fatigue damage degree. A small area fraction of martensite was observed in the austenite phase, and twin crystals were also present. As the fatigue stress increased, the area fraction of martensite slightly increased in the alloy damaged under 105 MPa fatigue stress. The area fraction of martensite remarkably increased in a case of 245 MPa since many of the austenite transformed to martensite by fatigue damage [1].

**Figure 2:**
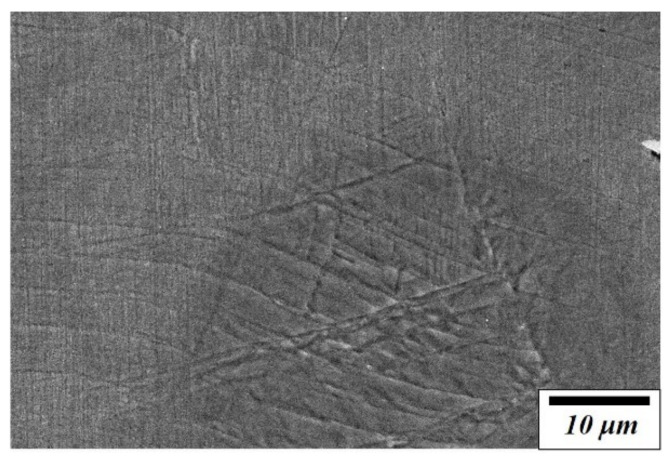
SEM image of Fe-22%Mn-12%Cr-4%Co-3%Ni-2%Si damping alloy damaged under 175-MPa fatigue stress.

Figure 2 shows an SEM image of a specimen damaged by 175-MPa fatigue stress. Microstructural observations confirmed the formation of martensite phases by fatigue stress, causing surface reliefs with a specific orientation and partially intersecting with each other [1,4]. Besides, the martensite produced due to fatigue stress is the same as that resulting from heat treatment and plastic deformation. In general, α’-martensite and ε-martensite were formed when the alloys under deformation-induced martensite transformation were deformed [3,5]. It is noted that ε-martensite is related to the damping capacity, whereas α’-martensite determines the mechanical properties [5]. Thus, it is important to clarify the effect of each martensite induced by fatigue stress on the mechanical properties and damping capacity.

**Figure 3:**
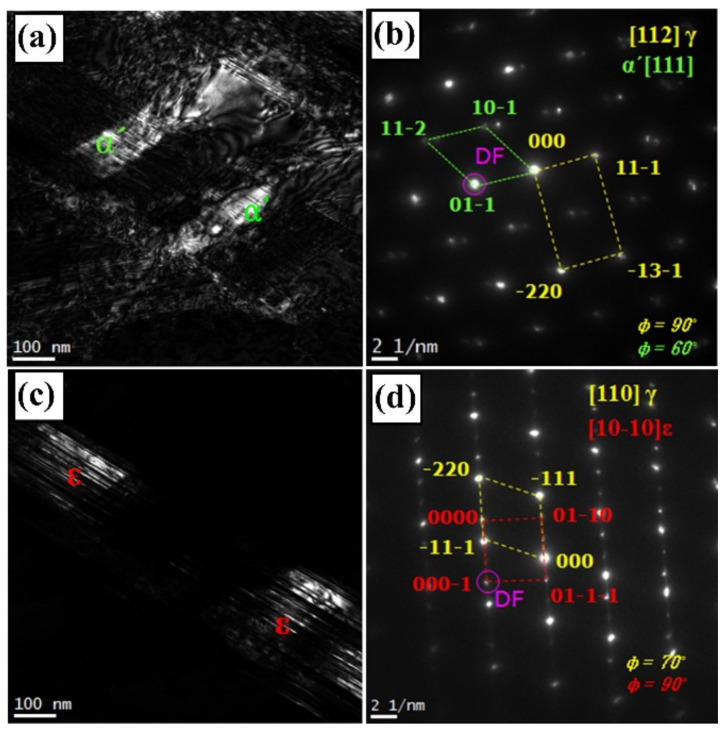
TEM images of martensite in the damaged Fe-22%Mn-12%Cr-4%Co-3%Ni-2%Si damping alloy under 175-MPa fatigue stress. (**a**) α′-martensite, (**b**) index of (**a**), (**c**) ε-martensite, and (**d**) index of (**c**).

Figure 3 shows TEM micrographs of dark-field image and SADP with its index for martensite formed in the damping alloy under 175-MPa fatigue stress. In general, for high-Mn alloys, α’-martensite forms on ε-martensite, whereas ε-martensite forms on austenite [13,14]. Herein, α’-martensite with the (111) plane formed directly on the (112) plane of the austenite phase (Figure 3b), whereas ε-martensite with the (100) plane formed on the (110) plane of the austenite phase (Figure 3d). This agrees well with the results in [15,16]. However, as the TEM observation shows, the α’-martensite formed at the intersection of ε-martensite was not identified and the austenite directly transformed to α’ martensite based on classic Bain correspondence [17]. This is a different result suggested by Olson-Cohen [13,18], demonstrating that the nucleation site of α’-martensite formed at the intersection (shear band) of ε-martensite by stress-assisted nucleation.

**Figure 4:**
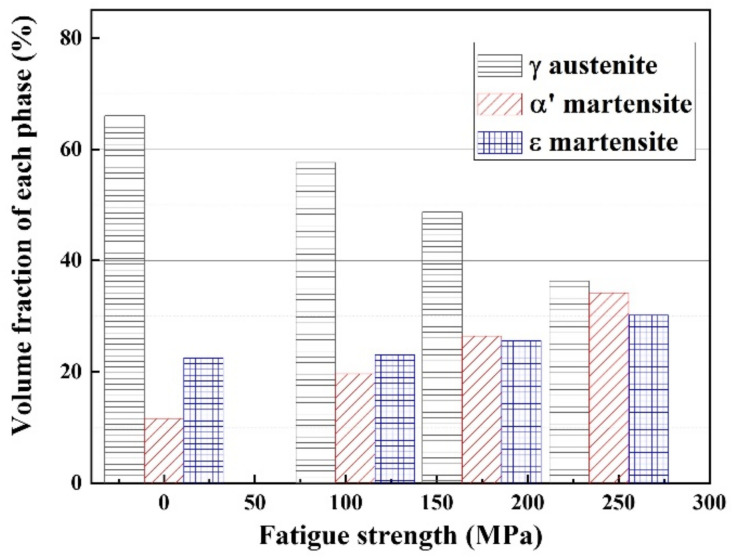
Effect of stress on the volume fraction of each phase in fatigue-damaged Fe-22%Mn-12%Cr-4%Co-3%Ni-2%Si damping alloy.

The volume fraction of each phase in the damaged damping alloy by fatigue stress of different magnitudes was evaluated (Figure 4). The alloy with no fatigue stress contains austenite, α’-martensite, and ε-martensite with 65%, 12%, and 23%, respectively. The volume fraction of austenite decreased, whereas that of α’- and ε-martensite increased since a volume fraction of the austenite transformed to α’- and ε-martensite as fatigue stress increased. As fatigue stress increased, the volume fraction of α′-martensite rapidly increased, but that of ε-martensite gradually increased. This variation is in good agreement with the result obtained by deformation-induced martensite transformation [19] in TRIP steels.

Depending on the component elements of alloys, the volume fraction of austenite and martensite can be varied. In specific, high Mn can lower the martensite-start temperature, stabilizing the ε-martensite [20], whereas the addition of Si [21,22,23] and Co [24] can effectively reduce the stacking fault energy (SFE), leading to an increase in the volume fraction of martensite.

**Figure 5:**
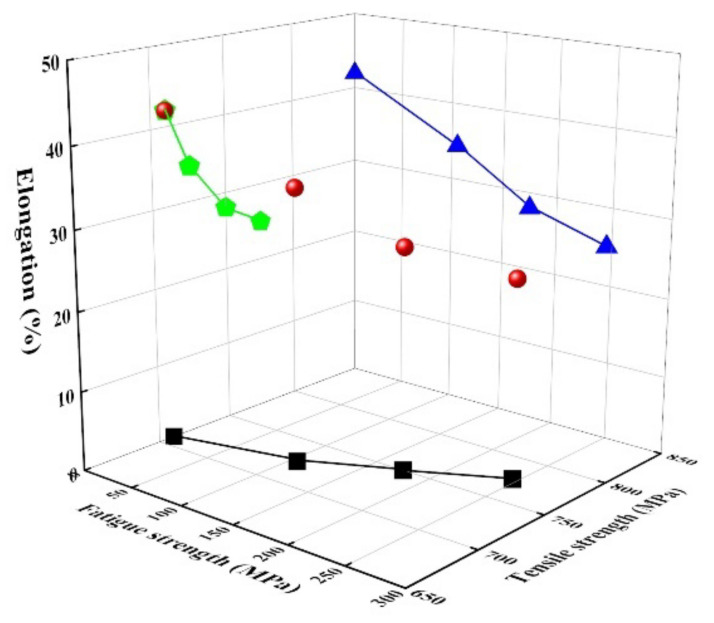
Mechanical properties of Fe-22%Mn-12%Cr-4%Co-3%Ni-2%Si damping alloy as a function of fatigue strength. Green pentagons: The relationship between elongation and tensile strength, blue triangles: The relationship between fatigue strength and elongation, and black squares: The relationship between fatigue strength and tensile strength.

Figure 5 shows the mechanical properties, tensile strength, and elongation in the alloys damaged by fatigue stress. As the fatigue stress increased, the tensile strength increased (black squares), whereas the elongation decreased (blue triangles). The tendency is because as the fatigue stress increased, the volume fraction of martensite increased (Figure 4). As expected, the elongation decreased with an increase in the tensile strength (green pentagons). Generally, for damping alloys, the mechanical properties are directly associated with the martensite transformation from austenite, which is in good agreement with our results.

However, the effect of martensite on the mechanical properties of alloys is not clarified since there is not much difference in volume fractions of each martensite. Further studies are needed to clarify the effect of α′- and ε-martensite on the mechanical properties in this alloy. Moreover, regarding the effect of element additions, Co, Ni, and Si in the Fe-Mn-Cr alloy on the mechanical properties, since Co [25] and Si [21] can attribute to a decrease of SFE, the formation of deformation-induced ε-martensite activates, and the increased fraction of martensite improved the tensile strength. In the case of Ni addition in this alloy, besides, it is well known that it is effective to lower the martensite start (Ms) temperature and stabilize the ε-martensite [20], attributing to increasing the tensile strength. In this alloy, the stacking fault energy ($\gamma_{sf}$) was calculated by [21] to be approximately 16.31 mJ/m^2^ (even though Co effect is exclusive), which is less than 20 mJ/m^2^, causing transformation-induced plasticity (TRIP).

**Figure 6:**
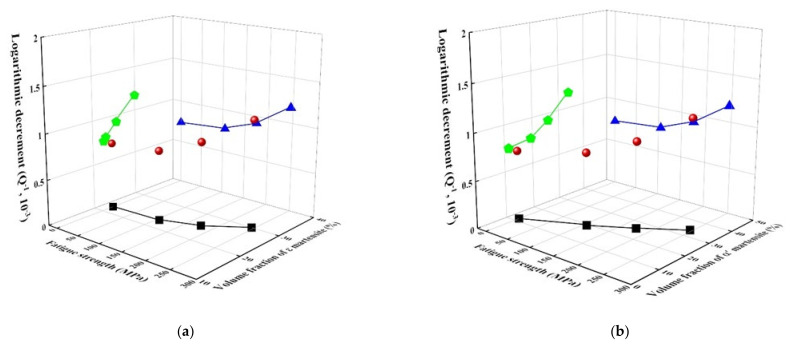
Damping capacity of fatigue-damaged Fe-22%Mn-12%Cr-4%Co-3%Ni-2%Si damping alloy. Green pentagons: The relationship between logarithmic decrement and volume faction of ε-martensite (or, α′-martensite), blue triangles: The relationship between logarithmic decrement and fatigue strength, and black squares: The relationship between fatigue strength and volume fraction of ε-martensite (or, α′-martensite). (**a**) Fatigue strength vs voulume fraction of ε-martensite vs logarithmic decrement ; (**b**) Fatigue strength vs voulume fraction of α′-martensite vs logarithmic decrement.

The damping capacity of the alloy was investigated to understand the relationship between the mechanical properties and damping capacity, as shown in Figure 6. As the fatigue stress increases, the damping capacity, which corresponds to logarithmic decrement, increases. This increase can be demonstrated by the experimental result that the volume fraction of ε martensite linearly increases as the fatigue strength increases [1,5]. It should be noted that ε martensite is more dominant in damping capacity than α’ martensite because the relationship between ε martensite and damping capacity is more linear (green pentagons) than that between α’ martensite and damping capacity. The well-known damping source can be suggested to the interface boundaries between austenite and ε-martensite, stacking fault boundaries in ε-martensite and variant boundary in martensitic plates in alloys [26] are related to ε-martensite. Accordingly, it is no wonder that the damping capacity has great linearity with the volume fraction of ε-martensite.

**Figure 7:**
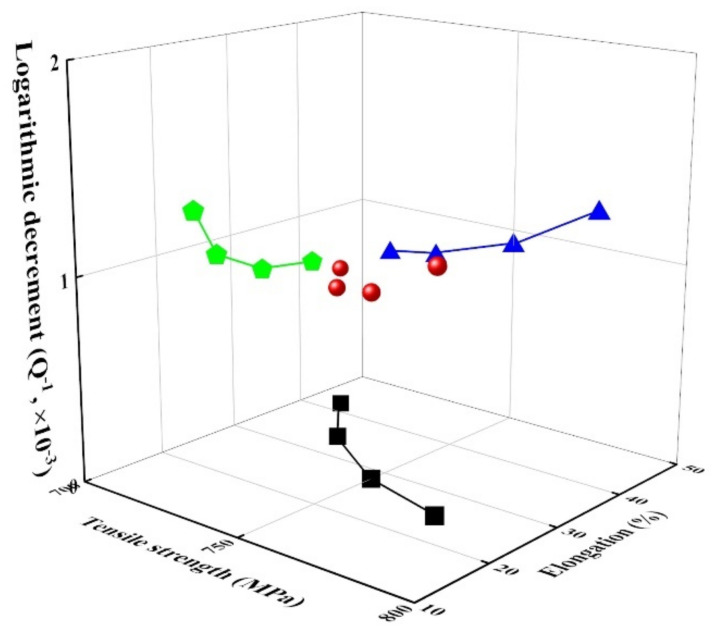
Relationship between the mechanical properties and logarithmic decrement in fatigue-damaged Fe-22%Mn-12%Cr-4%Co-3%Ni-2%Si damping alloy. Green pentagons: The relationship between logarithmic decrement and elongation, blue triangles: The relationship between logarithmic decrement and tensile strength, and black squares: The relationship between tensile strength and elongation.

Figure 7 shows the relationship between tensile strength (and elongation) and damping capacity of Fe-22%Mn-12%Cr-4%Co-3%Ni-2%Si damping alloys damaged by fatigue stress. The damping capacity gradually increased with an increase in tensile strength but decreased with an increase in elongation. This result is inconsistent with the general trend, in which the tensile strength is inversely proportional to damping capacity [27]. The increase in tensile strength and decrease in elongation as the fatigue stress increases in these alloys are attributed to the increased volume fraction of α′-martensite, whereas the damping capacity increases due to the increased volume fraction of ε-martensite [1,2].

## 4. Conclusions

We investigated the relationship between the tensile properties and damping capacity of Fe-22%Mn-12%Cr-4%Co-3%Ni-2%Si damping alloy damaged under different magnitudes of fatigue stress, and the following conclusions are drawn:

(1) Microstructural observations and X-ray analyses revealed the formation of α’- and ε-martensite in this damping alloy due to fatigue stress, causing surface relief with a specific orientation and partially intersecting with each other. TEM observation and pattern analysis reveal that both α′- and ε-martensites formed on the austenite, which is different from the Olson-Cohen model.

(2) The alloy with no fatigue stress contains austenite, α′-martensite, and ε-martensite with 65%, 12%, and 23%, respectively. As fatigue strength of the alloy increases, the volume fractions of α’- and ε-martensites increase.

(3) The tensile strength and damping capacity increase with an increase in the fatigue strength, whereas the elongation decreases.

(4) The addition of elements, Co, Ni, and Si in the alloy contributed to increasing the area fraction of martensite by lowering stacking fault energy and Ms temperature, resulting in the increase of the tensile strength.

(5) The increase in tensile strength and decrease in elongation as the fatigue stress increases in these alloys is attributed to the increased volume fraction of α′-martensite, whereas the damping capacity increases due to the increased volume fraction of ε-martensite. The relationship between the tensile properties and damping capacity in the fatigue-damaged damping alloy is inconsistent with the general trend for metals but agrees with that for alloys exhibiting a deformation-induced martensite transformation.

## Figures and Tables

**Table 1:** Chemical compositions (wt%).

C	N	P	S	Mn	Cr	Co	Ni	Si	Ti	Fe
0.01	0.1	0.01	0.01	22	12	4	3	2	0.3	Bal.

## Data Availability

Not applicable.
